# Cardioembolic Stroke: Clinical Features, Specific Cardiac Disorders and Prognosis

**DOI:** 10.2174/157340310791658730

**Published:** 2010-08

**Authors:** Adrià Arboix, Josefina Alió

**Affiliations:** a Cerebrovascular Division, Department of Neurology, Hospital Universitari del Sagrat Cor, Universitat de Barcelona, Barcelona, Spain; b CIBER de Enfermedades Respiratórias (CB06/06). Instituto Carlos III, Madrid; c Department of Cardiology, Hospital Universitari de Bellvitge, L’Hospitalet de Llobregat, Barcelona, Spain

**Keywords:** Cardioembolic stroke, recurrent embolization, atrial fibrillation, cardiac source of emboli, outcome.

## Abstract

This article provides the reader with an overview and up-date of clinical features, specific cardiac disorders and prognosis of cardioembolic stroke. Cardioembolic stroke accounts for 14-30% of ischemic strokes and, in general, is a severe condition; patients with cardioembolic infarction are prone to early and long-term stroke recurrence, although recurrences may be preventable by appropriate treatment during the acute phase and strict control at follow-up. Certain clinical features are suggestive of cardioembolic infarction, including sudden onset to maximal deficit, decreased level of consciousness at onset, Wernicke’s aphasia or global aphasia without hemiparesis, a Valsalva manoeuvre at the time of stroke onset, and co-occurrence of cerebral and systemic emboli. Lacunar clinical presentations, a lacunar infarct and especially multiple lacunar infarcts, make cardioembolic origin unlikely. The more common high risk cardioembolic conditions are atrial fibrillation, recent myocardial infarction, mechanical prosthetic valve, dilated myocardiopathy, and mitral rheumatic stenosis. Transthoracic and transesophageal echocardiogram can disclose structural heart diseases. Paroxysmal atrial dysrhyhtmia can be detected by Holter monitoring. In-hospital mortality in cardioembolic stroke (27.3%, in our series) is the highest as compared with other subtypes of cerebral infarction. In our experience, in-hospital mortality in patients with early embolic recurrence (within the first 7 days) was 77%. Patients with alcohol abuse, hypertension, valvular heart disease, nausea and vomiting, and previous cerebral infarction are at increased risk of early recurrent systemic embolization. Secondary prevention with anticoagulants should be started immediately if possible in patients at high risk for recurrent cardioembolic stroke in which contraindications, such as falls, poor compliance, uncontrolled epilepsy or gastrointestinal bleeding are absent.

## INTRODUCTION

Stroke is the leading cause of disability and the second most common cause of death worldwide [1,2]. Accurate definition of the mechanism of stroke is crucial as this will guide the most effective care and therapy. Cardioembolic stroke accounts for 14-30% of all cerebral infarctions [3-7]. In most cases, recurrence of cardioembolism can be prevented by oral anticoagulants. Therefore, for a patient with a cerebral infarct, early confirmation of a diagnosis of cardioembolic infarction is extremely important in order to initiate anticoagulation therapy for an adequate secondary prevention [8-12].

In the Sagrat Cor Hospital of Barcelona Stroke Registry, the frequency of cardioembolic stroke is 18% [13], a similar percentage than that in the studies of Bougousslavsky *et al*. [14] (16%) and Timsit *et al*. [15] (19.4%), higher than that reported by Vázquez *et al*. [16] (14%) and de Al-Rajed *et al*. [17] (14%), but lower than the percentages of Rothrock *et al*. [18] (22%) and Norrving and Löwenhielm [19] (30.6%). However, the incidence of cardioembolic stroke increases with age [13]. In the subgroup of patients younger than 65 years of age, cardioembolic stroke occurred in 14.6% of cases but in very old patients (age ≥ 85 years) cardioembolic stroke reached 36% of cases (Table **1**).

**Fig. (1):**
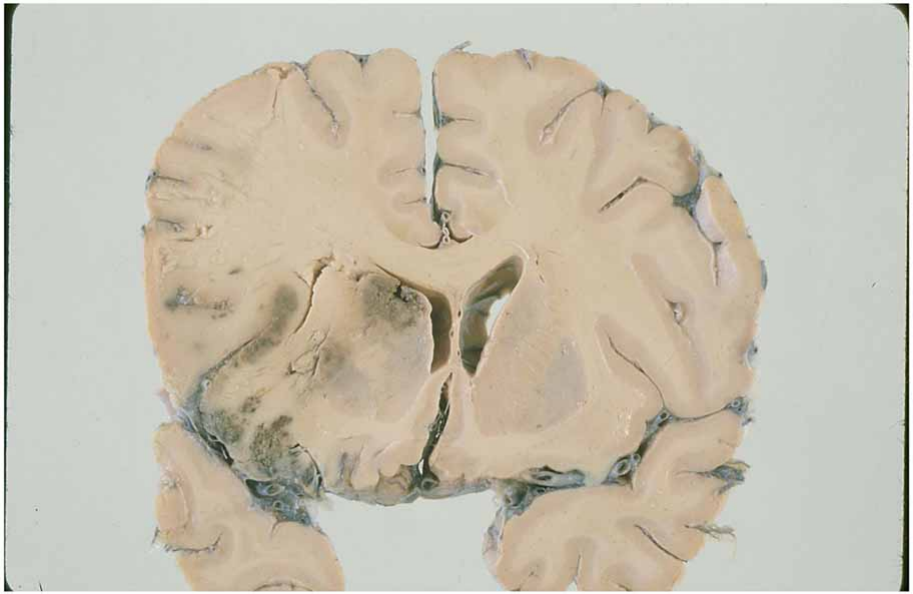
Histopathological specimen showing a hemorrhagic cerebral infarction of a cardioembolic origin with signs of ventricular displacement and brain herniation in the territory of the middle cerebral artery.

Embolism from the heart to the brain results from one of three mechanisms: blood stasis and thrombus formation in an enlarged (or affected by another structure alteration) left cardiac chamber (e.g., left ventricular aneurysm); release of material from an abnormal valvular surface (e.g., calcific degeneration); and abnormal passage from the venous to the arterial circulation (paradoxical embolism) [2]. Cardiac emboli can be of any size, but those of arising from the cardiac chambers are often large and hence especially likely to cause severe stroke, disability and death. Cardioembolic infarction is generally the most severe ischemic stroke subtype, with a low frequency of symptom-free at hospital discharge, a high risk of early and late embolic recurrences, and a high mortality [3,6] (Fig. **1**). 

There is no gold standard for making the diagnosis of cardioembolic stroke. The presence of a potential major cardiac source of embolism in the absence of significant arterial disease remains the mainstay of clinical diagnosis. When cardiac and arterial disease coexist (such as atrial fibrillation and ipsilateral carotid atheroma), determining the etiology of the ischemic stroke becomes more difficult. However, in many patients, history, physical examination, and routine diagnostic tests (electrocardiogram and findings on neuroimaging studies) are sufficient to easily make the diagnosis of most presumed cardiac emboligenic condition (e.g., atrial fibrillation, recent myocardial infarction, heart failure, prior rheumatic disease, splinter hemorrhages). An important exception is paroxysmal atrial fibrillation, which can be detected by 24-48 hour Holter monitoring immediately after stroke. Transthoracic echocardiogram can disclose structural cardiopathies (dilated cardiomyopathies, mitral stenosis and other structural ventricular diseases and intraventricular thrombus, vegetations or tumors) and enables measurement of the left atrial size and left ventricular systolic function [1,2]. Transesophageal echocardiogram is able to study the aortic arch and ascending aorta, left atrium and left atrial appendages, intra-arterial septum, pulmonary veins and valve vegetations [1,3]. Transesophageal echocardiography is more likely to be helpful in young patients with stroke, stroke of unknown cause and in patients with non-lacunar stroke. Cardiac magnetic resonance imaging (MRI) and nuclear cardiology studies (assessment of myocardial perfusion and analysis of ventricular function) may be useful in selected patients.

## CLINICAL FEATURES

Clinical features that support the diagnosis of cardioembolic stroke includes sudden onset to maximal deficit (< 5 min), which is present in 47-74% of cases and decreased level of consciousness at onset in 19-31% of cases [20,21]. In the study of Timsit *et al*. [22], altered consciousness was a predictive factor of cardioembolic infarction, with an odds ratio (OR) of 3.2 as compared with atherothrombotic infarction. Sudden onset of neurological deficit occurs in 79.7% of cases of cardioembolic stroke and in 38% of lacunar infarcts and in 46% of thrombotic infarctions (*P* < 0.01).

In 4.7-12% of cases, cardioembolic infarctions show a rapid regression of symptoms (the spectacular shrinking deficit syndrome) [23-26]. The recognition of this syndrome is important for a clinical suspicion of the cardioembolic origin of the cerebral infarction [26]. This dramatic improvement of an initially severe neurological deficit may be due to distal migration of the embolus followed by recanalization of the occluded vessel [27-29].

Wernicke’s aphasia or global aphasia without hemiparesis are other common secondary symptoms of cardioembolism [27,28]. In the posterior circulation, cardioembolism can produce Wallenberg’s syndrome, cerebellar infarcts, top-of-the basilar syndrome, multilevel infarcts, or posterior-cerebral-artery infarcts. Visual-field abnormalities, neglect, and aphasia are also more common in cardioembolic than in non-cardioembolic stroke. 

A classic cardioembolic presentation include onset of symptoms after a Valsalva-provoking activity (coughing, bending, etc.) suggesting paradoxical embolism facilitated by a transient rise in right atrial pressure and the co-occurrence of cerebral and systemic emboli [29]. 

On the other hand, other clinical symptoms classically associated with cardioembolic infarction, such as headache, seizures at onset [23] and onset during activity are not specific for cardioembolic stroke [4,27]. In addition, some signs or syndromes, such as lacunar clinical presentations (e.g., pure motor hemiparesis or ataxic hemiparesis), a lacunar infarct and particularly, multiple lacunar infarcts, make cardioembolic origin unlikely [30]. Cardiac embolism is a very rare cause of lacunar infarction (2.6-5% of cases) [31,32].

Neuroimaging data that support cardioembolic stroke include simultaneous or sequential strokes in different arterial territories. Owing to their large size, cardiac emboli flow to the intracranial vessels in most cases and cause massive, superficial, single large striatocapsular or multiple infarcts in the middle cerebral artery. Therefore, cardioembolic infarctions predominate in the carotid and middle cerebral artery distribution territories [28,29,33]. On the computed tomography (CT) scan, bihemispheric combined anterior and posterior circulation, or bilateral or multilevel posterior infarcts are suggestive of cardioembolism. MRI studies can increase the suspicion of cardio-embolism by demonstrating lesions not apparent on CT scans [1]. 

**Fig. (2):**
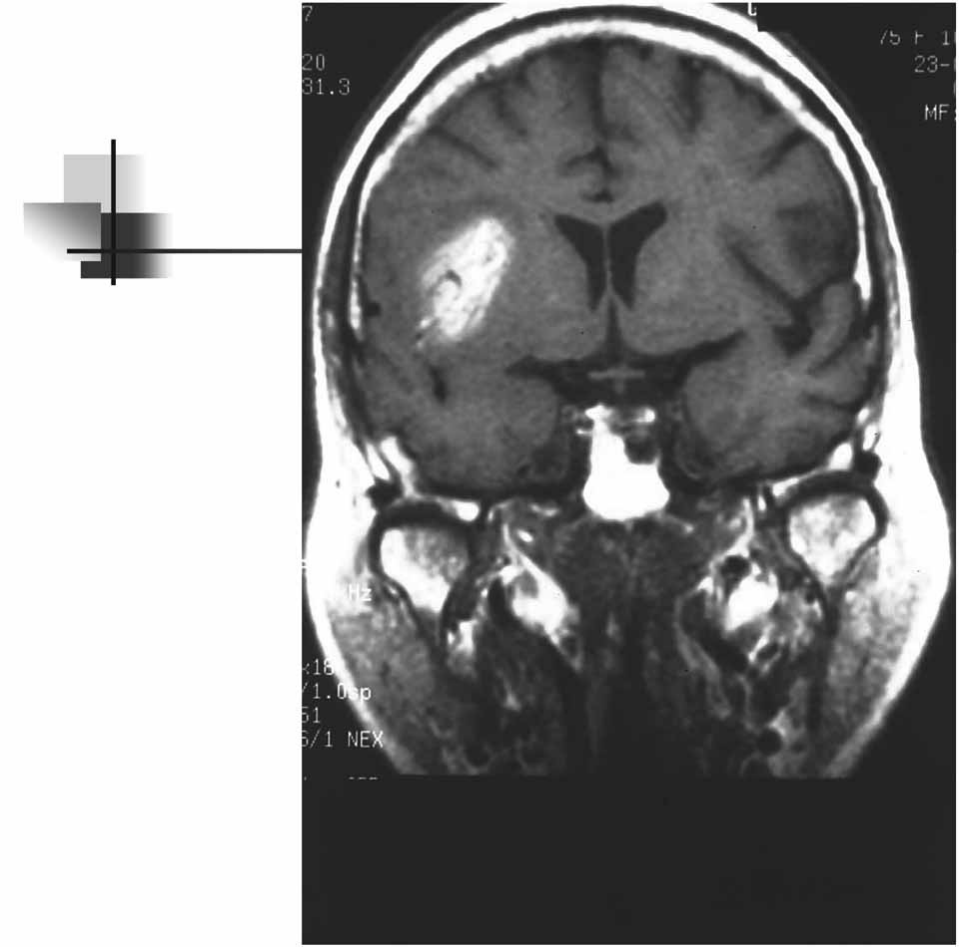
Hemorrhagic cardioembolic infarction in a patient with a spectacular shrinking deficit syndrome visualized in the brain MRI study (spin-echo hyperintensity T1-weighted image).

Hemorrhagic transformation of an ischemic infarct and early recanalization of an occluded intracranial vessel are suggestive of a cardiac origin of the stroke [1-3]. Hemorrhagic transformation occurs in up to 71% of cardioembolic strokes (Fig. **2**). As many as 95% of hemorrhagic infarcts are caused by cardioembolism. There are two types of hemorrhagic transformation: petechial or multifocal, which is normally asymptomatic and secondary hematoma, which has mass effects and clinical deterioration [34]. Secondary hematomas are unusual and are found in 0.8% of cases in our stroke registry [13]. The traditional explanation for hemorrhagic transformation is that the infarct is caused by blockage of a large artery by the thrombus; this blockage then causes local vascular spasm. Release of this local spasm and fragmentation of the thrombus allow the thrombus to migrate distally, exposing ischemic tissues and damaged vessel walls and capillaries to reperfusion. Arterial dissection at the site of impact of the thrombus is an alternative explanation.

Decreased alertness, total circulation infarcts, severe strokes (NIHSS >14), proximal middle cerebral artery occlusion, hypodensity in more than one third of the middle cerebral artery territory and delayed recanalization (> 6 hours after stroke onset) together with absence of collateral flow predict hemorrhagic transformation in acute cardioembolic stroke [1,4].

## SPECIFIC CARDIAC DISORDERS

A number of cardiac conditions have been proposed as potential sources of embolism. The risk of embolism is heterogeneous. The more common high risk cardioembolic conditions are atrial fibrillation, recent myocardial infarction, mechanical prosthetic valve, dilated myocardiopathy, and mitral rheumatic stenosis. Other major sources of cardioembolism include infective endocarditis, marantic endocarditis, and atrial myxoma. Minor sources of cardioembolism are patent foramen ovale, atrial septal aneurysm, atrial or ventricular septal defects, calcific aortic stenosis, and mitral annular calcification [5].

Atrial fibrillation is the most important cause of cardioembolic stroke [20,21]. Atrial fibrillation is the commonest sustained cardiac arrhythmia. Prevalence of atrial fibrillation increases with age, reaching a peak of 5% in people over 65 years of age, and both its incidence and prevalence are increasing. The disorder is associated with valvular heart disease, thyroid disorders, hypertension, and recent heavy drinking of alcohol. In Western populations, most causes of atrial fibrillation are unrelated to mitral valve disease. Instead, atrial fibrillation is now mainly secondary to ischemic or hypertensive heart disease. The attributable risk of stroke due to atrial fibrillation rises from 1.5% at the age of 50 to 24% at the age of 80. The incidence of stroke in people with non-valvular atrial fibrillation is estimated to be 2 to 7 times higher than in people without atrial fibrillation and for those with valvular atrial fibrillation, the risk is 17 times higher than that in age-matched controls. Chronic and recurrent atrial fibrillation appears to carry very similar stroke risk. Atrial fibrillation in the absence of organic heart disease or risk factors (lone atrial fibrillation) appears to carry significantly lower risk especially in younger patients (approximately 1.3% per year). Atrial fibrillation causes stroke because it leads to inadequate contraction of, and leads to stasis that is most marked in the left atrial appendage. Stasis is associated with increased concentrations of fibrinogen, D-dimer, and von Willebrand factor, which are indicative of a prothrombotic state, which in turn predisposes to thrombus formation with consequent increased rate of cerebral embolization [1]. In these patients, left ventricular dysfunction and left atrial size were independent echocardiographic predictors of later thromboembolism. Other factors associated with a particular high embolic risk are spontaneous echo contrast, left atrial thrombus or aortic plaque detected by transesophageal echocardiogram. Heart failure, hypertension, age > 75 years, and diabetes mellitus increase the risk of stroke in a more moderate but additive fashion [3].

The bradycardia-tachycardia (sick sinus) syndrome can be associated with cerebral embolic events.

**Fig. (3):**
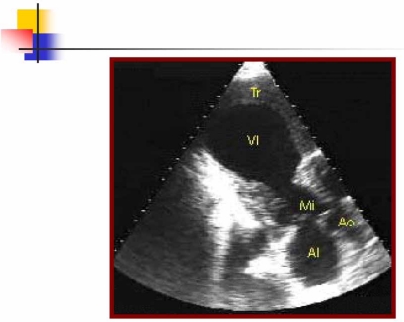
Transthoracic echocardiography shows a left ventricular aneurysm (VI) in a patient with history of acute myocardial infarction.

Approximately 2.5% of patients with acute myocardial infarction experience a stroke within 2 to 4 weeks of the infarction, and 8% of men and 11% of women will have an ischemic stroke within the next 6 years. Factors that enhance the risk of stroke include severe left ventricular dysfunction with low cardiac output, left ventricular aneurysm (Fig. **3**) or thrombus, and associated arrhythmias such as atrial fibrillation. Patients with an ejection fraction of less than 28% had a relative risk of stroke of 1.86 compared with patients with an ejection fraction greater than 35%. The incidence of early embolism is high, possibly up to 22% in the presence of a mural thrombus and is most likely when the thrombus is mobile or protrudes into the ventricle [6].

The annual rate of stroke in patients with congestive heart failure is 2%. The risk of stroke correlates with the severity of left ventricular dysfunction. Coexistent disease has a cumulative effect, and the combination of recent congestive heart failure and atrial fibrillation places the patient at particular high risk for cardioembolic stroke [2].

**Fig. (4):**
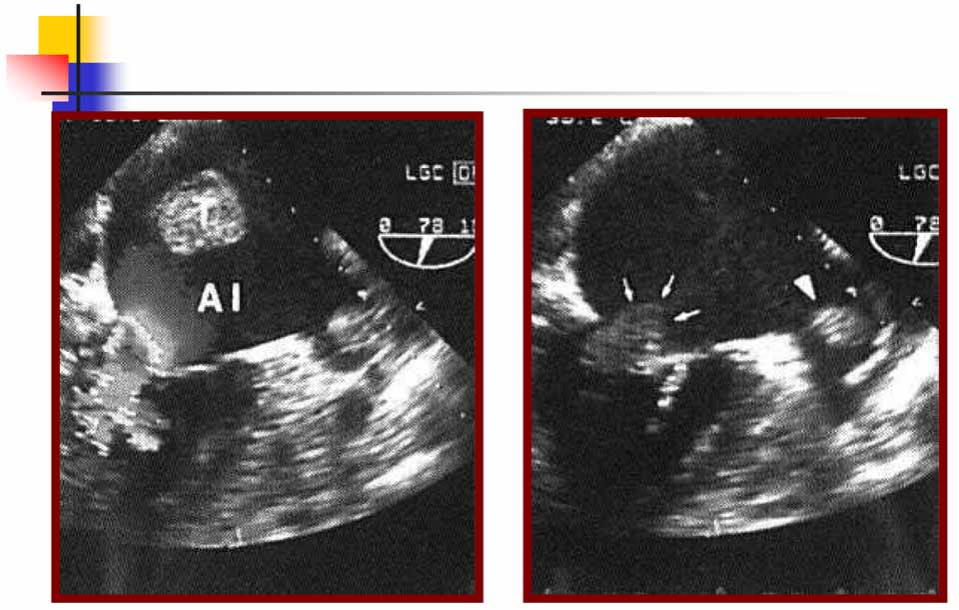
Transthoracic echocardiography reveals a thrombus in the left atrium (T) in a patient with double rheumatic mitral valve lesion and atrial fibrillation.

Rheumatic valvular heart disease (Fig. **4**) and mechanical prosthetic valves are well recognized risk factors for stroke even in the absence of documented atrial fibrillation. The two most commonly cited rheumatic valve abnormalities are mitral stenosis and calcific aortic stenosis [2]. 

**Fig. (5):**
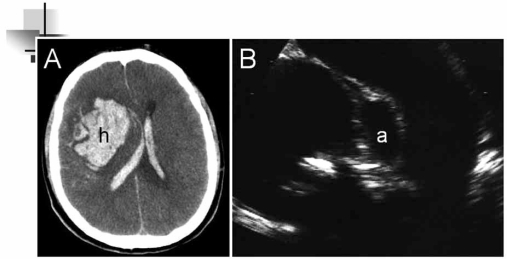
Right lobar hemorrhage (**A**) secondary to rupture of a mycotic aneurysm in the course of an infective bacterial endocarditis; transthoracic echocardiography (**B**) shows an abscess in the posterolateral aortic root (a) between the aortic valve leaflets and the mitral valve.

Two types of endocarditis, infective and non-infective, can cause stroke. Non-infective endocarditis can complicate systemic cancer, lupus, and the anti-phospholipid syndrome. Infective endocarditis is complicated by stroke in about 10% of cases. Most stroke happens early (before or during the first 2 weeks of appropriate antimicrobial therapy). Emboli can be multiple especially in the case of infection of prosthetic valves and in infections due to aggressive agents, such as *Staphylococcus aureus*. Mycotic aneurysm is an uncommon (1-5%) complication of infective endocarditis. They may also enlarge and rupture, which is fatal in many cases (Fig. **5**).

Myxomas account for more than half of primary cardiac tumors and thromboembolism is the most common presenting symptom in patients with myxomas. Other primary cardiac tumors include papillary fibroelastoma.

Patent foramen ovale and aortic arch atheroma are emerging embolic sources that are extensively described in other chapters.

Mitral annular calcification has been cited as a possible source of cerebral embolism with a relative risk of stroke of 2.1 in the Framingham Study independent of traditional risk factors for stroke [5]. In a recent study in patients with ischemic stroke of uncertain etiology, dense mitral annular calcification was an important marker of aortic arch atherosclerosis with high risk of embolism [35].

Spontaneous echo contrast is an independent echocardiographic risk factor for left atrial thrombus and its appendage and cardiac thromboembolic events. 

Cardiological substrate and pathophysiological mechanisms presumptively involved in cardioembolic stroke in the Sagrat Cor Hospital of Barcelona Stroke Registry [36] are shown in Table **2**. Atrial dysrhythmia without structural cardiac disease was documented in 89 (22%) patients, with a mean (SD) age of 75 (4) years (range 63–90 years). All these patients had normal electrocardiographic findings and 90% were asymptomatic. The cardiac condition associated with cardiogenic stroke was atrial fibrillation in 88 patients (chronic 67, paroxysmal 18, persistent 3) and atrial flutter in 1. A previous diagnosis of atrial dysrhythmia had been established in the outpatient setting in 51% of patients but none of the patients received anticoagulation.

Structural cardiac disease with sustained sinus rhythm was diagnosed in 81 (20%) of patients. Left ventricular systolic dysfunction was documented in 59 patients (ischemic heart disease in 35 and dilated cardiomyopathy in 24) associated with intraventricular thrombosis in 13. Other less frequent cardiac disorders included mitral annular calcification, cardiac tumors, aortic prosthetic valve, endocarditis, atrial septal aneurysm with patent foramen ovale, rheumatic mitral valve disease, mitral valve prolapse, calcified aortic stenosis with embolism during catheterization, and moderate mitral valve regurgitation.

In the remaining 232 (58%) patients, structural cardiac disorders were associated with atrial fibrillation in 230 cases and atrial flutter in 2. Hypertensive left ventricular hypertrophy was documented in 120 cases followed by rheumatic mitral valve disease in 49 cases and left ventricular dysfunction in 32 cases (ischemic heart disease in 19 and dilated cardiomyopathy in 13). Other less frequent cardiac disorders complicated with atrial fibrillation included mitral valve prolapse, mitral prosthesis, hypertrophic cardiomyopathy, lipomatous hypertrophy of the atrial septum, severe mitral regurgitation, and atrial septal aneurysm with patent foramen ovale.

The frequency of the different cardiac disorders in the overall series of 402 patients with cardioembolic stroke is shown in Table **3**. Atrial fibrillation was documented in 79.1% of patients (in association with structural cardiac disease in 72% of cases) followed by hypertensive left ventricular hypertrophy in 29.8% of patients, left ventricular dysfunction in 22.6%, rheumatic mitral valve disease in 12.4%, and mitral annular calcification in 9.9%. Mitral valve prolapse, atrial septal aneurysm with patent foramen ovale and degenerative heart valve disease were observed in only 1% of the patients. In the group of 118 patients with hypertensive left ventricular hypertrophy associated with atrial fibrillation, anteroposterior diameter of the left atrium was significantly larger than in the group of 88 patients with lone atrial fibrillation (45 ± 3 mm *vs.* 41 ± 3 mm, *P *< 0.001). On the other hand, 80.6% of these patients were asymptomatic, 50.5% had other vascular risk factor (cigarette smoking, diabetes mellitus, hyperlipidemia) besides hypertensive disease, and although a previous diagnosis of atrial dysrhythmia had been established in the outpatient setting in 43.7% of patients, none of the patients received anticoagulation at the time of stroke onset.

## MORTALITY OF CARDIOEMBOLIC INFARCTIONS

Cardioembolic infarctions are the subtype of ischemic infarcts with the highest in-hospital mortality during the acute phase of stroke [37-39]. In our experience and in agreement with the clinical series of Caplan *et al*. [34], the in-hospital mortality rate of cardioembolic infarction was 27.3% as compared with 0.8% for lacunar infarcts and 21.7% for atherothrombotic stroke (*P* < 0.01). Cardioembolic infarction is also associated with a lower rate of absence of functional limitation at discharge from the hospital, which may be related to the greater size of the lesion of cardioembolic stroke [15,22]. 

In a recent study carried out by our group in 231 patients with cardioembolic infarction with an in-hospital mortality rate of 27.3%, causes of death were as follows: a) non-neurological in 54% (n = 34), including pneumonia in 9, heart disease in 7, pulmonary thromboembolism in 7, sepsis in 5, sudden death in 4, and other causes in 2; b) neurological in 39.5% (n = 25), including brain herniation in 17, recurrence of cerebral ischemia in 6, and cerebral hemorrhage in 2; and of unknown cause in 6.5% (n = 4).

Early recurrent embolisms (within the first 7 days of stroke onset) were observed in 9 patients (3.9%) (peripheral embolisms in the extremities in 4, cerebral in 5). Only one patient was receiving therapeutic anticoagulation.

Mortality in patients with early embolic recurrence was 77.7% (7 of 9 cases) as compared with 25% for the remaining patients (*P* < 0.001). In the 5 patients with recurrent cerebral embolisms, the mortality rate was 100%. Two of the four patients with peripheral embolism died (mortality rate 50%).

In the multivariate analysis, four clinical variables were significantly associated with in-hospital mortality: age, congestive heart failure, hemiparesis, and decreased level of consciousness. However, when early recurrent embolism was added to the logistic regression model, this variable was associated with the highest risk for death (OR = 33.5).

Early and late embolic recurrences are not exceptional in cardioembolic infarction [38,40-42]. Recurrences are more frequent during the first days of stroke [10]. In the study of Sacco *et al*. [43], in which recurrences within the first 30 days were assessed, mortality was also significantly higher in the group of recurrences (20%) than in the group without recurrences (7.4%); survivors after stroke recurrence also showed a longer hospital stay. In the study of Yasaka *et al*. [44], mortality was also significantly higher in patients with recurrent embolism (19.6%) as compared with the remaining patients (8.8%).

Taking into account that in our series, only one patient with recurrent embolism was treated with therapeutic anticoagulation, we agree with Chamorro *et al*. [8] in the need of starting early prophylactic anticoagulation with sodium heparin in patients with cardioembolic infarction, with strict control of partial thromboplastin time (between 1.5 and 2) in order to prevent iatrogenic bleeding due to excessive anticoagulation.

## EMBOLIC RECURRENCE IN CARDIOEMBOLIC INFARCTION

The risk of early stroke recurrence in cerebral infarctions in general ranges between 1% to 10% according to the different series [38,40-42]. Some studies have shown that recurrences within the first 3 months are more common in cardioembolic infarction than in atherothrombotic infarcts. The risk of early embolic recurrence in cardioembolic stroke  varies between 1% and 22%. In the Cerebral Embolism Task Force, for example, it was estimated that around 12% of patients with cardioembolic infarctions would develop a second embolism within the first 2 weeks of the onset of symptoms [11]. In our experience, embolism recurrence during hospitalization occurred in 24 of 324 patients with cardioembolic stroke consecutively attended over a 10-year period (6.9% of cases) [45]. Embolic recurrence occurred within the first 7 days of neurological deficit in 12 patients (50%). The mean time of recurrence after stroke onset was 12 days. Recurrence of embolism within the first 30 days was observed in 5 of the 81 patients (6.1%) in the study of Yamanouchi *et al*. [46] in patients with cardioembolic infarction and non-valvular atrial fibrillation, in 6% of cerebral infarcts in the study of Sacco *et al*. [47], in 3.3% of patients from the Stroke Data Bank [43], and in 4.4% of patients included in the Lausanne Stroke Registry [48].

In our study, embolism recurrence was multiple in 3 cases (12.5%), which is consistent with data in the study of Yamanouchi *et al*. [46] in which 7 of 21 patients with cardioembolic infarctions had two or more stroke recurrences. The maximal risk of recurrence was the immediate period after the cardioembolic stroke.

Mortality in patients with recurrent embolism was two-fold higher as compared with the remaining patients (70.8% *vs *24.4%) [45], in agreement with the study of Sacco *et al*. [47] (19% *vs* 8%) in cerebral infarctions in general.

It is important to know factors associated with early embolic recurrence in cardioembolic infarction because patients in which these risk factors are present constitute a subgroup with the highest risk severity, requiring early treatment and strict medical control. However, risk factors for stroke recurrence are less known than risk factors for first-ever stroke. In our experience, alcohol abuse (OR = 21.8), hypertension with valvular heart disease and atrial fibrillation (OR = 4.3), nausea and vomiting (OR = 3.7), and previous cerebral infarct (OR = 3.2) were clinical predictors of cardioembolic stroke recurrence. In addition to these four variables, cardiac events (tachyarrhythmia, heart failure or acute myocardial infarction that occurred as medical complication during the patient’s hospital stay) were selected in the logistic regression model based on clinical, neuroimaging, and outcome variables (OR = 4.25). 

The association of hypertension with valvular heart disease and atrial fibrillation was a predictive variable of stroke recurrence but none of these variables was statistically significant when they were independently analyzed. In another study, valvular heart disease associated with congestive heart failure was the only predictive factor of stroke recurrence [49]. Although the presence of a structural cardiac disorder in a well known risk factor for system embolization [50,51], Lai *et al*. [52] also showed that patients with hypertension associated with non-valvular atrial fibrillation had a higher risk of embolic recurrence as compared to patients with only hypertension or with non-valvular atrial fibrillation only.

Involvement of cardiac center in the medulla oblongata may predispose to arrhythmias and cardiac arrest during the acute phase of stroke. Therefore, the presence of nausea and vomiting is a symptom usually associated with an infarction in the vertebrobasilar territory or progression compression of the brainstem due to an infarction in the carotid territory with transtentorial herniation, a clinical condition that can cause heart rhythm disturbances by concomitant involvement of the cardiac center and predispose to a potential cardioembolic recurrence [53-56].

In contrast to data observed in our study, the presence of a previous cerebral infarction was not a predictor of recurrence in the study of Sacco *et al.* [47]. However, other authors consider the presence of a cerebral infarction is one of the most powerful predictive factors recurrent embolism [54-58]. In the study of van Latum *et al*. [59], a previous thromboembolism of any kind was also a significant predictor of stroke recurrence.

Alcohol abuse was an important predictor of recurrent embolism in our experience of cardioembolic infarction [45], which is similar to that observed in the study of Sacco *et al*. [47]. There is evidence of a strong relationship between stroke and alcohol: a) alcohol intoxication is a risk factor for cerebral infarction [60]; b) a higher frequency of alcohol abuse among stroke patients has been demonstrated [61-63]; c) other studies even claim that continued alcohol abuse is a true risk factor for stroke [64-67]. In Caucasian populations, "J-shaped" relationship has been documented between the protective effect of mild daily alcohol consumption and an increase in the risk of cerebral infarction by increasing daily alcohol consumption [61-63]. Although its effect on cardioembolic stroke is still unclear, there are several pathophysiological mechanisms by which alcohol can cause stroke [61,68-79]. These include the following:

- favoring hypertension, increasing platelet aggregation, plasma osmolarity, hematocrit, and erythrocyte aggregation and deformability;
- a consequence of a dilated cardiomyopathy due to alcohol abuse;
- induction of cardiac arrhythmias (atrial fibrillation, ventricular extrasystoles, junctional tachycardia, paroxysmal supraventricular tachycardia, and ventricular tachycardia in subjects who are habitual alcohol consumers, in sporadic alcohol users, and in those abstaining from alcohol [72]. Ethanol also increases adrenal release of catecholamines, which predisposes to arrhythmogenicity; in addition, acetaldehyde –a major alcohol metabolite- is also arryhthmogenic [73,64].
- changes in the cerebral blood flow and autoregulation in relation to alcohol abuse have been also reported;
- liver disease secondary to alcohol abuse [68].

Our study therefore suggest that alcohol abuse is an important independent factor associated with embolic recurrence in cardioembolic stroke

Any of the mechanisms outlined above may predispose to a new embolism, although the presence of a non-ischemic cardiomyopathy associated with the possibility of cardiac arrhythmia are probably the more common potential mechanisms.

A classification system based on independent risk factors for stroke and used in clinical practice for predicting stroke in patients with non-valvular atrial fibrillation is the CHADS2 index [80] (acronym for Congestive heart failure, Hypertension, Age, Diabetes mellitus and stroke). CHADS2 is formed by assigning 1 point each for the presence of congestive heart failure, hypertension, age 75 years or older, and diabetes mellitus, and by assigning 2 points for history of stroke or transient ischemic attack. Those patients with CHADS2 score of 0 or 1 have a low annual risk of stroke (1%), CHADS2 score of 2 identifies patients with moderate risk (annual risk of 2.5%), and patients with a score of 3 or greater are estimated to have a high risk of stroke (annual risk > 5%).

Early embolism is the main independent risk factor for in-hospital mortality in patients with cardioembolic infarction [40].Timing of initiation of anticoagulant treatment remains an area of uncertainty, since there is concern regarding exacerbating the risk of hemorrhage into regions of infarction (”hemorrhagic transformation”) after ischemic stroke. Guidelines propose arbitrary deferral of anticoagulation for 2 weeks in patients hospitalized with stroke by extrapolation from acute trials with full-dose heparin, where reduced early recurrent ischemic stroke is balanced by increased hemorrhagic risk. In patients with transient ischemic attack or minor stroke and with exclusion of cerebral hemorrhage, oral anticoagulation can be initiated within 3-5 days. However, we agree with Chamorro et al. [8] that secondary prevention with anticoagulants should be started immediately if possible in high recurrent embolic cardioembolic stroke risk patients without contraindications, such as falls, poor compliance, uncontrolled epilepsy, or gastrointestinal bleeding. Thus, contrary to the recommendation to delay anticoagulation in patients with extensive cardioembolic infarction or marked neurological deficit, immediate anticoagulation may be indicated in this subpopulation of cardioembolic infarction with maximal risk for early cardioembolic recurrence. According to Yasaka *et al.* [44], early anticoagulation with intravenous sodium heparin reduces the frequency of recurrent events and would reduce mortality, providing that it is initiated as soon as possible and maintaining activated thromboplastin time values below twice the control values. Oral anticoagulation with warfarin would be indicated later.

## EARLY DIFFERENTIAL DIAGNOSIS BETWEEN CARDIOEMBOLIC AND ATHEROTHROMBOTIC INFARCTS 

Clinical data exclusive for cardioembolic infarctions or atherothrombotic infarctions are lacking. However, to establish an early diagnosis of cardioembolic infarction may have a therapeutic interest. In a study of our group [81], it was shown that atrial fibrillation and sudden onset of neurological symptoms were independent clinical factors significantly associated with cardioembolic stroke, whereas hypertension, chronic obstructive pulmonary disease, diabetes, dyslipemia, and age were clinical variables independently associated with atherothrombotic infarction.

On the other hand, clinical data traditionally related to cardioembolic infarction, such as seizures or headache, were not predictors of cardioembolic stroke, which is consistent with results of the studies of Ramirez-Lassepas *et al*. [82], Kittner *et al*. [83,84], and Caplan *et al*. [20].

## ATRIAL FIBRILLATION IN CARDIOEMBOLIC AND ATHEROTHROMBOTIC INFARCTIONS 

Atrial fibrillation is the main cardiac disorder in the different series of cardioembolic infarctions from industrialized countries reported in the literature [27,37,85]. However, atrial fibrillation can be also observed in atherothrombotic infarcts, not as an embolic etiology but a marker of other conditions that lead to ischemic stroke, such as atherosclerosis. It may be therefore considered as an epiphenomenon or a clinical manifestation of atherosclerotic disease [50]. In this respect, not all cerebral infarctions in patients with atrial fibrillation are of cardioembolic origin [21]. In our study, atrial fibrillation was diagnosed in 16.5% of patients with thrombotic occlusion or arterial stenosis grater than 70% presumably responsible for the cerebral infarction [86]. In these cases, some clinical or echocardiographic findings related to cardioembolism, such as recent congestive heart failure or increase of the left atrial size, or left ventricular dysfunction were absent [87,88]. Bogousslavsky *et al*. [21] showed that 76% of patients with cerebral infarcts in the carotid vascular territory with atrial fibrillation, the presumable pathophysiological mechanism of stroke was cardioembolic since a significant arterial vascular disease could not be documented. However, in 11% of the cases, the presumable mechanism was atherosclerosis because severe arterial stenosis or occlusion correlated with clinical features, and in the remaining 13%, the cerebral infarct could be explained by occlusion of small perforating arterial vessels in association with hypertension.

Accordingly, in a patient with cerebral infarction and atrial fibrillation it is important to make an early and precise diagnosis of the subtype of cerebral infarct, although the differential diagnosis between cardioembolic and atherothrombotic stroke with atrial fibrillation may be difficult to establish at the onset of neurological deficit. In recent classifications of stroke subtypes, this distinction is not made and these patients are included in the subgroup of cerebral infarctions of undetermined cause due to the simultaneous presence of two potential etiologies [89]. However, it should be noted that using the results of appropriate neurological and cardiological studies carried out in a delayed during hospitalization, in most of the cases, it is possible to establish the correct classification of stroke in the definite nosological entity [20].

In our experience based on 2000 patients with acute cerebrovascular disease [86], 1712 (85.6%) had a cerebral infarction. A total of 347 (17.4%) were classified as cardioembolic infarction, 452 (22.6%) as atherothrombotic infarction. Patients with cardioembolic infarction and atrial fibrillation accounted for 76.6% of the cases (n = 226), and patients with atherothrombotic infarction and atrial fibrillation for 16.5% (n = 75).

### Cardioembolic Infarctions with and without Atrial Fibrillation

When patients with cardioembolic infarction with and without atrial fibrillation were compared, female sex, history of heart failure, sudden onset of neurological deficit, altered consciousness, motor, sensory and visual deficits, and parietal topography of the ischemic lesion were more frequently recorded in cardioembolic stroke patients with atrial fibrillation. Coronary heart disease, smoking, and topography of the infarct in the internal capsule were more frequent in cardioembolic stroke patients without atrial fibrillation. The in-hospital mortality rate was 31.6% in patients with atrial fibrillation and 14.8% in those without atrial fibrillation (*P* < 0.01) [86].

### Atherothrombotic Infarctions with and without Atrial Fibrillation

In the comparison of patients with atherothrombotic infarction with and without atrial fibrillation, those with atrial fibrillation were older, with a predominance of females, and a higher frequency of coronary and valvular heart disease, sudden onset of neurological deficit, sensory and visual deficits, speech disturbance, parietal, temporal, and occipital topography, and infarction in the vascular territory of the middle cerebral artery. Cardiac events were also more frequent. In atherothrombotic infarcts without atrial fibrillation, smoking, involvement of the cranial nerves and vertebral vascular topography were more common. Absence of functional dysfunction on hospital discharge was also more frequent. The in-hospital mortality rate was 29.3% in patients with atrial fibrillation and 18.8% in those without atrial fibrillation (*P* < 0.04) [86].

### Cardioembolic Infarctions and Atherothrombotic Infarctions with Atrial Fibrillation

When predictors of cardioembolic infarction or atherothrombotic infarction with atrial fibrillation were assessed in the multivariate analysis, rheumatic valve disease (OR = 4.6) and sudden onset of symptoms (OR = 1.8) were independently associated with cardioembolic stroke, whereas subacute stroke onset (OR = 8.01), chronic obstructive pulmonary disease (OR = 5.2), hypertension (OR = 3.6), dyslipemia (OR = 2.6), and diabetes (OR = 2.26) were independently associated with atherothrombotic infarction [86].

It should be noted that atrial fibrillation had a negative effect on outcome, both in cardioembolic and atherothrombotic infarction. It has been hypothesized that the worse outcome associated with atrial fibrillation may be explained by a higher prevalence of heart failure and ischemic heart disease. This hypothesis coincides in part with our results, given that a higher occurrence of heart failure in patients with cardioembolic stroke and a higher frequency of ischemic heart disease in patients with atherothrombotic stroke were observed. This may contribute to a decrease in cerebral blood flow as cerebral autoregulatory mechanisms in the ischemic area are impaired [90]. Other authors suggest that chronic atrial fibrillation may cause a significant reduction of regional blood flow [91], which may normalize when sinus rhythm is attained after successful cardioversion [92]. Other studies indicate that an increase in mortality may be explained by the more advanced age of the patients, a higher volume of the lesion, or a higher initial intensity of focal neurological deficit in patients with atrial fibrillation [93,94]. In summary, cerebrovascular disease in ischemic cardioembolic or atherothrombotic infarct is more severe in the presence of atrial fibrillation as compared to patients with normal sinus rhythm.

## Figures and Tables

**Table 1.** Distribution of Cerebral Infarctions According to Age in the Sagrat Cor Hospital of Barcelona Stroke Registry

Subtype of Cerebral Infarction (n = 1840)	Years of Age			
	< 65 (n= 314)	65–74 (n=501)	75–84 (n=722)	≥ 85 (n=303)
Cardioembolic	46 (14.6)	100 (20)	213 (29.5)	109 (36)
Atherothrombotic	66 (21.0)	159 (31.7)	233 (32.3)	95 (31.4)
Lacunar	93 (29.6)	159 (31.7)	173 (24)	59 (19.5)
Unknown cause	61 (19.4)	69 (13.8)	81 (11.2)	37 (12.2)
Unusual cause	48 (15.3)	14 (2.8)	22 (3.0)	3 (1)

Percentages in parenthesis.

**Table 2:** Cardiac Disorders and Pathophysiological Mechanisms Presumptively Associated with Cardioembolic Stroke in 402 Patients. Distribution by Cardiac Source Risk Groups. Sagrat Cor Hospital of Barcelona Stroke Registry

Cardiac Source of Embolism	Total Patients	
Arrhythmia without structural heart disease	89 (22.1%)	
Atrial fibrillation		88
Atrial flutter		1
Isolated structural heart disease	81 (20.1%)	
Ischaemic heart disease		35
Acute myocardial infarction		3 (thrombus 2)
Left ventricular aneurysm		7 (thrombus 3)
Left ventricular ejection fraction < 40%		12
Akinesia/dyskinesia ≥ two segments		13 (thrombus 3)
Dilated cardiomyopathy		24 (thrombus 5)
Mitral annular calcification		14^*^
Cardiac tumour		4
Aortic prosthetic valve		4
Endocarditis		2
Atrial septal aneurysm with patent foramen ovale		2
Rheumatic mitral valve disease		1
Mitral valve prolapse		1
Calcified aortic stenosis		1
Moderate mitral valve regurgitation		1
Structural heart disease and atrial arrhythmia	232 (57.7%)	
Atrial fibrillation		230
Atrial flutter		2
Hypertrophic hypertensive cardiac disease		120
Rheumatic mitral valve disease		49 (thrombus 7)
Ischaemic heart disease		19
Left ventricular aneurysm		3 (thrombus 1)
Left ventricular ejection fraction < 40%		9
Akinesia/dyskinesia ≥ two segments		7 (thrombus 1)
Mitral annular calcification		26^†^
Dilated cardiomyopathy		13 (thrombus 2)
Mitral valve prolapse		4
Mitral prosthetic valve		3 (thrombus 2)
Lipomatous hypertrophy of the atrial septum		2
Hypertrophic cardiomyopathy		2
Atrial septal aneurysm and patent foramen ovale		2
Severe mitral regurgitation		2

* In 8 patients in association with a structural cardiac source of embolism (dilated cardiomyopathy, n=2; ischaemic heart disease with ventricular ejection fraction < 40%, n=2; acute myocardial infarction, n=1; left ventricular aneurysm, n =1; aortic prosthetic valve, n=1; mitral leaflet calcification with moderate regurgitation, n=1).

† In 10 patients in association with a structural cardiac source of embolism (hypertensive left ventricular hypertrophy, n=8; mitral leaflet calcification with severe degenerative type regurgitation, n=2).

**Table 3:** Frequency of the Different Cardiological Substrate in 402 Patients with Cardioembolic Stroke in the Sagrat Cor Hospital of Barcelona Stroke Registry

Cardiac Source of Embolism	Total Patients	
Atrial fibrillation	318 (79.1%)	
Lone atrial fibrillation		88
Associated with structural cardiac disease		230
Hypertensive left ventricular hypertrophy	120 (29.8%)	
Associated with atrial fibrillation		118
Associated with atrial flutter		2
Left ventricular systolic dysfunction	91 (22.6%)	
Sinus rhythm		59
Atrial fibrillation		32
Rheumatic mitral valve disease	50 (12.4%)	
Mitral annular calcification	40 (9.9%)	
Mitral valve prolapse	5 (1.2%)	
Atrial septal aneurysm with patent foramen ovale	4 (1%)	
Degenerative heart valve disease	4 (1%)	
